# Linking first lactation survival to milk yield and components and lactation persistency in Tunisian Holstein cows

**DOI:** 10.5194/aab-62-153-2019

**Published:** 2019-04-04

**Authors:** Marwa Grayaa, Sylvie Vanderick, Boulbaba Rekik, Abderrahman Ben Gara, Christian Hanzen, Siwar Grayaa, Rodrigo Reis Mota, Hedi Hammami, Nicolas Gengler

**Affiliations:** 1 Institut National Agronomique de Tunisie, Tunis, 1082, Tunisia; 2 TERRA Teaching and Research Centre, Gembloux Agro-Bio Tech, University of Liège, Gembloux, 5030, Belgium; 3 Département des Productions Animales, Ecole supérieure d'Agriculture de Mateur, Mateur, 7030, Tunisia; 4 Clinical Department of Production Animals, Faculty of Veterinary Medicine, University of Liège, Liège, 4000, Belgium

## Abstract

Genetic parameters were estimated for first lactation survival defined as a binary trait (alive or dead to second calving) and the curve shape traits of milk yield, fat and protein percentages using information from 25 981 primiparous Tunisian Holsteins. For each trait, shape curves (i.e. peak lactation, persistency), level of production adjusted to 305 days in milk (DIMs) for total milk yield (TMY), and average fat (TF %) and protein (TP %) percentages were defined. Variance components were estimated with a linear random regression model under three bivariate animal models. Production traits were modelled by fixed herd × test-day (TD) interaction effects, fixed classes of 25 DIMs × age of calving × season of calving interaction effects, fixed classes of pregnancy, random environment effects and random additive genetic effects. Survival was modelled by fixed herd × year of calving interaction effects and age of calving × season of calving interaction effects, random permanent environment effects, and random additive genetic effects. Heritability ($h^{2}$) estimates were 0.03 (±0.01) for survival and 0.23 (±0.01), 0.31 (±0.01) and 0.31 (±0.01) for TMY, TF % and TP %, respectively. Genetic correlations between survival and TMY, TF % and TP % were 0.26 (±0.08), -0.24 (±0.06) and -0.13 (±0.06), respectively. Genetic correlations between survival and persistency for fat and protein percentages were -0.35 (±0.09) and -0.19 (±0.09), respectively. Cows that had higher persistencies for fat and protein percentages were more likely not to survive.

## 1 Introduction

The cow's life is divided into two distinct periods: a non-productive period, from birth to first calving, and a productive period from first calving to death or slaughter. The latter period is commonly called productive lifetime or herd life (Jenko et al., 2013). Herd life can also be defined as the cumulated days in lactation or as the number of lactations (Jairath et al., 1994). It is closely related to the concept of survival to certain point of life, such as subsequent calving or a certain age (Van Pelt et al., 2016). It results from a combination of characteristics directly associated with the ability of the cow to remain in the herd (Tsuruta et al., 2005; Ahlman et al., 2011), i.e. being able to calve normally, while resisting metabolic disorders, diseases such as mastitis, infertility or lameness, and producing enough and good-quality milk (Weigel, 2006). There are many reasons for a dairy farmer to wish for animals that survive well. The most important is economic benefit even though achieving a long herd life is still a major challenge. In Belgium, it was reported that less than one-third of the cows reached the fourth lactation (Gengler et al., 2005). In Canada and the Netherlands, 25 % and 13 % of primiparous cows left the herd during their first lactation, respectively (Jairath et al., 1998; Van Pelt et al., 2016). Ajili et al. (2007) reported that in Tunisia more than 57 % of cows were culled after the first two lactations, and only 7 % of them reached their fifth lactation. This is a situation that has fundamentally not changed in recent years.

Animal breeding can be a tool to improve cow survival since it is additive and cumulative. However, the selection and breeding decisions have to be made early in a cow's life although survival information is only available at the end of life. That makes selection for survival difficult. A crucial element is the modelling of the dairy cow's survival. Two extreme types of models are used. The first type is proportional hazard function based on survival models (Ducrocq et al., 1988; Ducrocq and Sölkner, 1994). These models are defined as single-trait as they model survival from day to day (Ducrocq and Sölkner, 1994). The second type is multi-trait models in which the survival to a fixed point in the productive life (e.g. each lactation) is a different trait (Jairath et al., 1998). Equivalent random regression implementations of the latter strategy were proposed by Gengler et al. (2005) and Van Pelt et al. (2016). By considering fixed points in the life of dairy cows (e.g. alive or dead to second calving), these models represent longevity as survival to that point. These models have the major advantage that survival is genetically no longer considered to be the same trait during the whole productive life. This is a valid hypothesis given the fact that the culling risk is higher in later parities than in earlier parities (De Vries et al., 2010). Furthermore, it has been reported that correlations of lactation survival between parities are different from unity (Jairath et al., 1998; Gengler et al., 2005; Van Pelt et al., 2015). Given this situation, survival may be defined as a binary trait and, therefore, in theory, should be fitted by a non-linear model (Gianola, 1982). Nevertheless, no clear advantage of using univariate non-linear over linear models was reported for binary or even categorical traits (Matos et al., 1997; Phocas and Laloë, 2003; Vanderick et al., 2014). However, bivariate linear-threshold models may show greater advantages than bivariate linear–linear models (Varona et al., 1999; Ramirez-Valverde et al., 2001), but they require greater computations and rely on specific assumptions. Therefore, most countries use linear models in their national genetic evaluation routine for lactation survival although such data violate the assumption of normality (Interbull, 2017).

The correlation estimates between early indicator traits and survival traits are of great interest. A long history of studies has linked survival to milk yield (Pool et al., 2003; Ajili et al., 2007). More specifically, M'hamdi et al. (2010) reported that survival was mainly influenced by milk yield in Tunisia. These authors did not address in detail the relationships between survival and a more detailed description of the milk yield across lactation (lactation curve shape and milk composition). To our knowledge, there are few studies linking survival to the shape of the lactation curve (Reents et al., 1996; Cole and Null, 2009). In the past, there were some efforts to make use of pre-established lactation curve shape traits, i.e. by defining the lactation persistency as the ability to maintain constant yield during lactation (Gengler, 1996) or in the context of linking lactation curve shape to disease traits (Harder et al., 2006). These efforts could not be considered a success since persistency definitions are much more diverse and since estimated correlations suffered from the lack of control by other variations (e.g. overall level of production) as mentioned by Gengler (1996). Therefore, the aim of the present study was to link the lactation curve shape of milk yield (represented as level and persistency) and major milk components (fat and protein percentage level and persistency) to survival of first lactation in primiparous Tunisian Holstein cattle.

## 2 Material and methods

### 2.1 Data

Pedigree and daily milk yield (MY), fat percentage (F %) and protein percentage (P %) test-day (TD) records of 25 981 primiparous Tunisian Holstein cows that participated in the official milk recording in the period from 2000 to 2014 were provided by the Tunisian Genetic Improvement Center (Tunisian Livestock and Pasture Office). The animals came from 34 herds located in the north (24), central (3) and eastern regions (7) of Tunisia. Each herd consisted of at least 50 cows in order to keep informative herds (Van Pelt et al., 2016). Cows aged less than 20 or more than 42 months at first calving were excluded. Ages at calving were divided into five classes: <=25; 26 to 27; 28 to 29; 30 to 31; and >31 months. Seasons of calving were separated into four classes: fall (from September to November), winter (from December to February), spring (from March to May) and summer (from June to August). The pregnancy status was defined by gestation month: first to ninth (n=9 classes). Test-day records of daily MY < 3 and >80 kg, F % < 1.5 and >9 %, and P % < 1 and >7 % were excluded following Hammami et al. (2008), who worked on similar data. Test-day records between 5 and 305 days in milk (DIMs) remained for further analyses. Lactations with the first TD exceeding 82 days from calving were deleted. Only cows having at least three TDs were kept. Pedigree information was traced back five generations, resulting in a pedigree file with 1054 sires of which 202 had more than 30 daughters. Descriptive statistics of the edited dataset are presented in Table 1.

**Table 1:** Descriptive statistics.

Trait	Mean	SD	Minimum	Maximum
Milk yield (n=150955)				
Number of test-day records per lactation	6.23	1.32	3.00	8.00
Milk yield test-day records	20.70	7.50	3.10	6.90
Fat percentage (n=121208)				
Number of test-day records per lactation	4.93	1.75	3.00	8.00
Fat percentage test-day records	3.38	0.71	1.51	9.00
Protein percentage (n=130769)				
Number of test-day records per lactation	5.39	1.60	3.00	8.00
Protein percentage test-day records	3.12	0.39	1.01	7.00

### 2.2 Trait definition

*Persistency and peak*. In this study, persistency was defined following Jamrozik et al. (1997) as the difference between the values at DIM 280 (i.e. MY280 – MY at DIM 280; F280 – F % at DIM 280; P280 – P % at DIM 280) and at the peak (i.e. MY60 – MY at DIM 60; F60 – F % at DIM 60; P60 – P % at DIM 60).

*Level of production*. These traits were defined as total MY (TMY) and average F % (TF %) and P % (TP %) over 305 DIMs.

*Lactation survival*. The survival trait was defined as a binary trait (alive or dead to second calving). We have chosen to analyse survival during first lactation, due to the rapid availability of information for survival and the lower complexity of data modelling. Moreover, a high genetic correlation with third-lactation survival was found (results not shown). Similar results were also reported by Gengler et al. (2005). A cow was considered to have survived its first lactation if a cow had at least one TD from the second lactation and was coded as 2. A cow was considered to be dead if she did not have a subsequent lactation and was coded as 1. Finally, the cows studied inevitably had a first lactation but not necessarily a second lactation because they could leave the herd before calving again. Since TD records used in this study were taken until the end of 2014 and the occurrence of a second calving was checked up to 21 October 2017, we considered that cows had sufficient opportunity to calve again. Given the fact that there is little inter-farm commerce of older cows in Tunisia we expect that this way of procedure generates reliable survival values.

### 2.3 Model

Three bivariate analyses (MY, P % or F % TD with survival) using a linear random regression model were performed. The model was written with matrix notation as follows:

1 $$y=Xb+Q\left(Za+Zp\right)+e,$$

where y is a vector of MY, F % or P % and survival; b is a vector of fixed effects, herd × TD, classes of 25 DIMs × age of calving × season of calving and pregnancy for production traits, and herd × year of calving and age of calving × season of calving for survival; a and p are vectors of random regression coefficients for additive genetic and permanent environment effects, respectively; Q is a matrix for the modified second-order Legendre polynomials (constant = 1; linear and quadratic) for production traits and for the null-order Legendre polynomial (constant = 1) for survival; X, Z are incidence matrices linking observations with respective effects, and e is a vector of residuals.

The covariance matrix structure of the model is as follows:

2 $$\left[\begin{array}{c} a\\ p\\ e \end{array}\right]=\left[\begin{array}{ccc} A⊗\mathrm{Ka} & 0 & 0\\ 0 & I⊗\mathrm{Kp} & 0\\ 0 & 0 & R \end{array}\right],$$

where Ka is a 4×4 (co)variance matrix of the additive genetic random regression coefficients; A is the additive relationship matrix among all animals; Kp is the 4×4 (co)variance matrix of the random permanent environment regression coefficients; I is an identity matrix having as dimension the number of animal with records, and R is a 2×2 diagonal matrix of residual variance. The genetic variance matrix among all DIMs and production traits was obtained as following:

3 $$G={\mathrm{QKaQ}}^{\prime }.$$

For all traits, additive genetic variance ($\sigma_{a}^{2}$), permanent environment variance ($\sigma_{p}^{2}$), residual variance ($\sigma_{e}^{2}$), phenotypic variance ($\sigma_{T}^{2}$) and cow-specific variance ($\sigma_{c}^{2}=\sigma_{a}^{2}+\sigma_{p}^{2}$) were obtained by the appropriate functions of regression coefficients Ka and Kp.

The variances at the peak and late lactation for MY, F % and P % were computed at DIM 60 and DIM 280 by using the estimated (co)variances for those DIMs. The variances for persistency were calculated as follows:

4σa2=QpKaQp′,5σp2=QpKpQp′,

where $Q_{p}=Q_{280}-Q_{60}$; $Q_{60}$ and $Q_{280}$ are vectors of three modified Legendre polynomials associated with DIM 60 and DIM 280, respectively.

**Figure 1:**
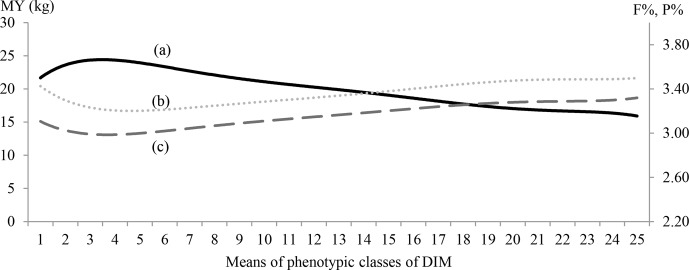
MY (continuous line; **a**), F % (dotted line; **b**) and P % (dashed line; **c**) by 25 classes of days in milk (DIMs).

The variances for level of production, TMY, TF % and TP % were the variances derived from the sums of full Legendre polynomial coefficients summed from DIM 1 to DIM 305 of each trait.

Heritability ($h^{2}$) estimates of survival as well as MY, F % and P % at DIM 60 and DIM 280 were defined as the ratio of $\sigma_{a}^{2}$ to $\sigma_{T}^{2}$. In order to take into account the fact that TMY, TF %, TP % and persistency are, following Jamrozik et al. (2001), second-stage traits defined from test days, the $h^{2}$ estimates were computed as the ratio of $\sigma_{a}^{2}$ and $\sigma_{c}^{2}$.

Random effects were assumed to be normally distributed and residual variances were assumed to be independent and constant along lactation. The use of a constant residual variance was also chosen so as not to render the model even more complex.

To avoid environmental covariances being considered as genetic covariances, within an animal, survival was modelled by a permanent environment effect, as proposed by Bastin et al. (2012). This effect together with a diagonal residual matrix allowed us to model non-genetic covariances between test-day yields, with the permanent environment effect part modelling the non-genetic cow-specific correlations between lactation-curve-based traits as level of production, persistency and survival. The sum of genetic and non-genetic cow-specific effects was considered as the total cow-specific effect and used in the study.

Variance components were estimated using the REMLF90 software (Misztal et al., 2002). After convergence of the REMLF90, the average information REML (AIREML) software was run using final estimates of the REMLF90. The standard error (SE) of (co)variance estimates and $h^{2}$ were obtained using the average information REML matrix (Misztal et al., 2014). Approximated SE for correlations and $h^{2}$ were calculated using equations provided by Falconer and Mackay (1996) and Rustin et al. (2009).

## 3 Results

From studied cows, only 75.20 % of cows reached their second lactation. The quarter of cows that left the herd during their first lactation had partial lactation data. Indeed, 35.34 % of the latter cows left the herd in the first 100 DIMs, 19.87 % left the herd in the middle of lactation (between 100 and 200 DIMs) and 44.79 % left the herd during the last part of lactation (after 200 DIMs).

Means for 25-DIM classes of MY, F % and P % are shown in Fig. 1.

The MY lactation curve had an inverted shape compared with content curves. The MY curve showed a typical shape: the peak occurred between the third and fourth classes (DIM 50–60 post-partum) and gradually fell thereafter. Curves for F % and P % were characterized by an early decrease reaching nadir point between DIM 35 and 50 after calving, followed by a steady increase to the end of lactation. Highest values for F % (3.5 %) were observed at the beginning and at the end of lactation (DIM 305) while the highest P % value (3.3 %) was reached at the end of lactation (DIM 305).

**Table 2:** Heritability (in bold), additive genetics (above the diagonal), permanent environment correlation (below the diagonal), cow-specific correlation (in brackets) between first lactation survival and MY60, MY280, F60, F280, P60, P280, TMY, TF %, TP % and P as well as associated standard errors.

	Survival	MY60	MY280	TMY	Persistency
Survival	0.03±0.01	0.21±0.10	0.20±0.10	0.26±0.08	-0.01±0.09
MY60	0.12±0.01 (0.17±0.01)	0.14±0.01	0.33±0.05	0.87±0.01	-0.53±0.03
MY280	0.18±0.07 (0.25±0.01)	0.58±0.01 (0.52±0.01)	0.16±0.01	0.73±0.02	0.62±0.03
TMY	0.16±0.01 (0.23±0.01)	0.89±0.01 (0.89±0.01)	0.88±0.01 (0.84±0.01)	0.23±0.01	-0.07±0.03
Persistency	0.07±0.01 (0.09±0.01)	-0.45±0.01 (-0.47±0.01)	0.46±0.01 (0.51±0.01)	-0.01±0.01 (-0.02±0.01)	0.21±0.01
	Survival	F60	F280	TF %	Persistency
Survival	0.03±0.01	-0.07±0.29	-0.40±0.14	-0.24±0.06	-0.35±0.09
F60	0.05±0.03 (0.04±0.03)	0.02±0.01	0.21±0.22	0.68±0.05	-0.33±0.07
F280	-0.05±0.03 (-0.10±0.02)	0.15±0.12 (0.16±0.08)	0.05±0.01	0.80±0.02	0.85±0.07
TF %	-0.02±0.01 (-0.05±0.01)	0.73±0.02 (0.71±0.01)	0.76±0.01 (0.77±0.01)	0.31±0.01	0.41±0.01
Persistency	-0.08±0.02 (-0.11±0.01)	-0.63±0.06 (-0.54±0.01)	0.67±0.05 (0.74±0.01)	0.0±0.03 (0.17±0.01)	0.05±0.01
	Survival	P60	P280	TP %	Persistency
Survival	0.03±0.01	0.04±0.16	-0.23±0.12	-0.13±0.06	-0.19±0.09
P60	-0.01±0.03 (-0.01±0.08)	0.04±0.01	-0.31±0.11	0.59±0.04	-0.73±0.04
P280	-0.07±0.03 (-0.09±0.02)	0.35±0.09 (0.05±0.01)	0.08±0.01	0.56±0.03	0.87±0.04
TP %	-0.05±0.01 (-0.06±0.01)	0.79±0.01 (0.71±0.01)	0.79±0.01 (0.69±0.01)	0.31±0.01	0.10±0.03
Persistency	-0.05±0.02 (-0.07±0.01)	-0.45±0.08 (-0.59±0.01)	0.67±0.05 (0.78±0.01)	0.14±0.03 (0.11±0.01)	0.08±0.01

DIMs: days in milk; MY60: milk yield at DIM 60; MY280: milk yield at DIM 280; F60: fat percentage at DIM 60; F280: fat percentage at DIM 280; P60: protein percentage at DIM 60; P280: protein percentage at DIM 280; TMY: total milk yield production; TF %: total fat percentage production; TP %: total protein percentage production; ±: standard errors.

**Figure 2:**
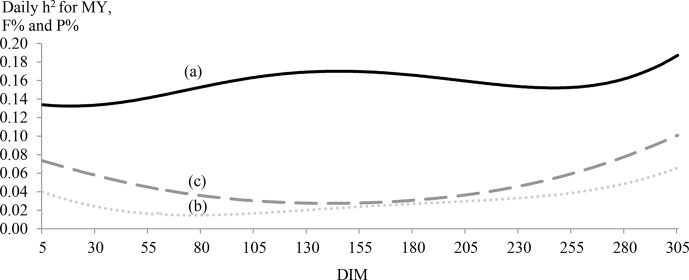
Daily estimates of heritability ($h^{2}$) for MY (continuous line; **a**), F % (dotted line; **b**) and P % (dashed line; **c**) by days in milk (DIMs).

Additive genetic, permanent environment and cow-specific correlation estimates among all traits, $h^{2}$ estimates as well as all standard errors are shown in Table 2. Genetic correlations of survival were positive for TMY (0.26±0.08) and negative for TF % (-0.24±0.06) and TP % (-0.13±0.06). On the other hand, permanent environment and cow-specific correlations were, respectively, 0.16 (±0.01) and 0.23 (±0.01) between survival and TMY, -0.02 (±0.01) and -0.05 (±0.01) between survival and TF %, and -0.05 (±0.01) and -0.06 (±0.01) between survival and TP %. Genetic correlations between survival and MY persistency not to be seemed different from zero (-0.01±0.09) whereas permanent environment and cow-specific correlations were positive but low (0.07±0.01 and 0.09±0.01, respectively). Nevertheless, genetic correlations between survival and persistency were moderate and negative for F % (-0.35±0.09) and for P % (-0.19±0.09). Permanent environment and cow-specific correlations were close to zero between survival and persistency for MY, F % and P % except for F % where cow-specific correlation between survival and persistency was 0.11 (±0.01). Genetic correlation between survival and MY (MY60 and MY280) was positive and had the same magnitude at peak lactation (MY60) and in late lactation (MY280). For permanent environment and cow-specific correlations, a different trend was observed and correlations were higher in late lactation (Table 2). The genetic correlation estimates between survival and F % were negative and of a high magnitude in late lactation (-0.40±0.14). Genetic correlation of P % with survival seemed not to be significantly different from zero for P60 (0.04±0.16) and was -0.23 (±0.12) for P 280.

Daily estimates of heritability for MY, F % and P % by DIM were plotted in Fig. 2. Milk yield was more heritable at the middle and end of lactation. For P % and F %, trends were different and showed border effects. The lowest heritability estimates for F % were observed between the 55th and the 95th DIM, while the lowest estimates were observed between the 111th and the 172nd DIM. Generally, daily heritability estimates of F % were small and did not even exceed 0.06 for two-thirds of lactation.

Heritability estimates for survival ranged from 0.02 to 0.04 while those of persistency were 0.21 (±0.01) for MY, 0.05 (±0.01) for F % and 0.14 (±0.01) for P % (Table 2). Estimates of $h^{2}$ for TMY, TF % and TP % were 0.23 (±0.01), 0.31 (±0.01) and 0.31 (±0.01), respectively.

## 4 Discussion

The proportion of cows with partial lactation found in this study confirmed the findings of M'hamdi et al. (2010) on the same population, who reported that the highest risk of culling was found for cows at the beginning and at the end of the first lactation.

Lactation curve shapes for MY and components plotted in this study (Fig. 1) were similar to those of Bouallegue et al. (2014), who found similar curve shapes in a Mediterranean hot climate.

Few studies have used random regression models to investigate the relationship between milk yield and survival whereas studies linking major milk components to survival are even scarcer (Dematawewa and Berger, 1998; Zavadilovà and Zink, 203). The literature provided evidence that survival was influenced by MY (Pool et al., 2003; Ajili et al., 2007; M'hamdi et al., 2010). Genetic correlations between TMY and survival are very variable as survival's definition changes from a continuous trait to survival at certain point of time (Dematawewa and Berger, 1998; Pool et al., 2003). However, these correlations are usually positive as also found in the current study. Positive phenotypic and cow-specific correlations between survival and TMY seemed to indicate that in Tunisia farmers may provide better management for highly producing cows during their first lactation and eventually prioritize their health treatment over other cows in order to keep them in the herd for as long as possible. On the other hand, the genetic antagonism found between survival and TF % was higher than in the findings of Dematawewa and Berger (1998) and Zavadilová and Zink (2013), who reported genetic correlations of -0.20 and -0.18, respectively. These same authors reported similar genetic correlations between survival and TP % (-0.15 and -0.13, respectively). This antagonism can partly be explained by the fact that high F % and lower P % require a constant high consumption of energy, and consequently cows may undergo an excessive mobilization of reserves leading to a negative energy balance. This negative energy balance is associated with fertility and therefore with the ability to achieve a next calving and therefore to survive.

According to our results, cows that had higher F % and P % persistency, are expected to exit the herd earlier. The question that arises is whether this negative genetic relationship is due to the beginning of lactation or not. Unexpectedly, results showed that it is rather due to the end of lactation. A hypothesis could be, that lower MY leads to lower survival, and at the same time lower MY leads to higher F % and P %. This could finally end up with a negative correlation of F280 to survival. However, survival was not genetically associated with F % and P % at their peak. Morton et al. (2017) reported that low levels of P % in early lactation can be due to an energy deficit that results in reproductive difficulties. At this early stage of lactation, farmers seem to postpone the culling decision by giving the cow more chances to overcome its reproductive problems. This may explain weak environmental and cow-specific correlations in early lactation found in our study.

As regards survival, $h^{2}$ estimates were similar to other studies using a sire model (Boettcher et al., 1999; Du Toit et al., 2009) and lower than the majority of the results of survival models (Boettcher et al., 1999). Milk yield persistency had similar $h^{2}$ compared to those found by Canaza Cayo et al. (2015) (ranged from 0.10 to 0.33). For the persistency of F % and P %, $h^{2}$ estimates were low. Given that $h^{2}$ of persistency varied significantly depending on definition (Biassus et al., 2010), it could be stated that persistency's definition was not optimal for this trait as it was for MY and for P %. However, the SE obtained in this study showed that these estimates of $h^{2}$ differ from zero. Therefore, these low estimates could be due to the stressful climatic conditions, constrained feeding resources and the sampling process preventing the full genetic potential of cows from being expressed (Hammami et al., 2008). Milk contents and detailed milk composition (fatty acids and metabolites) were found to be more sensitive to warmer conditions compared to the milk yield at a phenotypic and genetic level (Hammami et al., 2015). Tunisia experiences high temperatures and humidity (THI > 70) going beyond the thermoneutral threshold for nearly half the year.

For TMY, TF % and TP %, $h^{2}$ estimates were higher than those found by Hammami et al. (2008) by using a random regression model (0.17, 0.13 and 0.15, respectively). However, $h^{2}$ daily estimates were low, especially for F % and P %. These results were similar to those found by Hammami et al. (2008) on the same population.

## 5 Conclusion

In conclusion, the results of the present study revealed a positive genetic, environmental and cow-specific relationship between survival and TMY. Moreover, survival was genetically negatively correlated to TF % and slightly less to TP %. The most likely reasons could be that highly (milk) yielding cows are preferentially treated; however, high fat and protein production through high percentages requires a constant high consumption of energy. Consequently, cows may undergo an excessive mobilization of reserves that can lead to a negative energy balance that is associated with the ability of the cow to calve again.

On the other hand, correlations between survival and MY persistency were low. However, cows that had higher F % persistency were more likely to exit the herd earlier. The question that arose was whether this negative relationship was due to the beginning of lactation. Unexpectedly, results showed that it was linked to high percentages at the end of lactation.

## Data Availability

The data (pedigree and phenotypic data) cannot be made publicly accessible because they are not the property of the authors but that of the Genetic Improvement Center (Tunisian Livestock and Pasture Office) acting on the behalf of the owners of the performance-recorded cows.

## References

[bib1.bib1] Ahlman T, Berglund B, Rydhmer L, Strandberg E (2011). Culling reasons in organic and conventional dairy herds and genotype by environment interaction for longevity. J Dairy Sci.

[bib1.bib2] Ajili N, Rekik B, Ben Gara A, Bouraoui R (2007). Relationships among milk production, reproductive traits, and herd life for Tunisian Holstein-Friesian cows. Afr J Agr Res.

[bib1.bib3] Bastin C, Berry DP, Soyeurt H, Gengler N (2012). Genetic correlations of days open with production traits and contents in milk of major fatty acids predicted by mid-infrared spectrometry. J Dairy Sci.

[bib1.bib4] Biassus IO, Cobuci JA, Costa CN, Rorato PRN, Braccini Neto J, Cardoso LL (2010). Persistence in milk, fat and protein production of primiparous Holstein cows by random regression models. Rev Bras Zootecn.

[bib1.bib5] Boettcher PJ, Jairath LK, Dekkers JCM (1999). Comparison of methods for genetic evaluation of sires for survival of their daughters in the first three lactations. J Dairy Sci.

[bib1.bib6] Bouallegue M, M'Hamdi N, Ben Hamouda M, Haddad B (2014). Study of non-genetic factors on the shape of lactation curves for milk yield, fat and protein percents of Holstein-Friesian cows under hot Mediterranean climate. Archivos de Zootecnia.

[bib1.bib7] Canaza-Cayo AW, Lopes PS, da Silva MV, de Almeida Torres R, Martins MF, Arbex WA, Cobuci JA (2015). Genetic parameters for milk yield and lactation persistency using random regression models in Girolando cattle. J Anim Sci.

[bib1.bib8] Cole JB, Null DJ (2009). Genetic evaluation of lactation persistency for five breeds of dairy cattle. J Dairy Sci.

[bib1.bib9] Dematawewa CMB, Berger PJ (1998). Genetics and breeding genetic and phenotypic parameters for 305-day yield, fertility, and survival in Holsteins. J Dairy Sci.

[bib1.bib10] De Vries A, Olson JD, Pinedo PJ (2010). Reproductive risk factors for culling and productive life in large dairy herds in the eastern United States between 2001 and 2006. J Dairy Sci.

[bib1.bib11] Ducrocq V, Sölkner J (1994). The Survival Kit-a Fortran package for the analysis of survival data.

[bib1.bib12] Ducrocq V, Quaas RL, Pollak EJ, Casella G (1988). Length of productive life of dairy cows: 1. Justification of a Weibull Model. J Dairy Sci.

[bib1.bib13] Du Toit J, van Wyk JB, Maiwashe A (2009). Genetic parameter estimates for functional herd life for the South African Jersey breed using a multiple trait linear model. S Afr J Anim Sci.

[bib1.bib14] Falconer DS, Mackay TFC (1996). Genetic and environmental correlations. Introduction to Quantitave Genetics.

[bib1.bib15] Gengler N (1996). Persistency of lactation yields: a review.

[bib1.bib16] Gengler N, Vanderick S, Mayeres P, Gillon A, Croquet C (2005). Genetic evaluation of cow survival using a lactation random regression model, Interbull Bulletin no. 33.

[bib1.bib17] Gianola D (1982). Theory and analysis of threshold characters. J Anim Sci.

[bib1.bib18] Hammami H, Rekik B, Soyeurt H, Ben Gara A, Gengler N (2008). Genetic parameters for Tunisian Holsteins using a test-day random regression model. J Dairy Sci.

[bib1.bib19] Hammami H, Vendenplas J, Vanrobays ML, Rekik B, Bastin C, Gengler N (2015). Genetic analysis of heat stress effects on yield traits, udder health and fatty acids of Wallon Holstein cows. J Dairy Sci.

[bib1.bib20] Harder B, Bennewitz J, Hinrichs D, Kalm E (2006). Genetic parameters for health traits and their relationship to different persistency traits in German Holstein dairy cattle. J Dairy Sci.

[bib1.bib21] (2017). Description of National Genetic Evaluations Systems for dairy
cattle traits as applied in different Interbull member countries [Internet].

[bib1.bib22] Jairath LK, Hayes JF, Cue RI (1994). Multitrait restricted maximum likelihood estimates of genetic and phenotypic parameters of lifetime performance traits for Canadian Holsteins. J Dairy Sci.

[bib1.bib23] Jairath L, Dekkers JCM, Schaeffer LR, Liu Z, Burnside EB, Kolstad B (1998). Genetic evaluation for herd life in Canada. J Dairy Sci.

[bib1.bib24] Jamrozik J, Schaeffer LR, Dekkers JCM (1997). Genetic evaluation of dairy cattle using test day yields and random regression model. J Dairy Sci.

[bib1.bib25] Jamrozik J, Gianola D, Schaeffer LR (2001). Bayesian estimation of genetic parameters for test day records in dairy cattle using linear hierarchical models. Livest Prod Sci.

[bib1.bib26] Jenko J, Gorjanc G, Kovač M, Ducrocq V (2013). Comparison between sire-maternal grandsire and animal models for genetic evaluation of longevity in a dairy cattle population with small herds. J Dairy Sci.

[bib1.bib27] Matos CA, Thomas DL, Gianola D, Perez-Enciso M, Young LD (1997). Genetic analysis of discrete reproductive traits in sheep using linear and non-linear models: II. Goodness of fit and predictive ability. J Anim Sci.

[bib1.bib28] M'hamdi N, Aloulou R, Bouallegue M, Brar SK, Ben Hamouda M (2010). Study on functional longevity of Tunisian Holstein dairy cattle using a Weibull proportional hazard model. Livest Prod Sci.

[bib1.bib29] Misztal I, Tsuruta S, Strabel T, Auvray B, Druet T, Lee DH (2002). BLUPF90 and related programs (BGF90).

[bib1.bib30] Misztal I, Tsuruta S, Lourenço D, Aguilar I, Legarra A, Vitezica Z (2014). Manual for BLUPF90 family of programs.

[bib1.bib31] Morton JM, Auldist MJ, Douglas ML, Macmillan KL (2017). Milk protein concentration, estimated breeding value for fertility, and reproductive performance in lactating dairy cows. J Dairy Sci.

[bib1.bib32] Phocas F, Laloë D (2003). Evaluation models and genetic parameters for calving difficulty in beef cattle. J Anim Sci.

[bib1.bib33] Pool MH, Olori VE, Calus MPL, Veerkamp RF (2003). Aspects of milk yield adjustment in the parameter estimation for genetic evaluation of survival. Interbull Bulletin n30.

[bib1.bib34] Ramirez-Valverde R, Misztal I, Bertrand JK (2001). Comparison of threshold *vs* linear and animal *vs* sire models for predicting direct and maternal genetic effects on calving difficulty in beef cattle. J Anim Sci.

[bib1.bib35] Reents R, Reinhardt F, Abramowsky M (1996). Calculation of persistency proofs from the German multi-lactation model for production traits. Interbull Bulletin n 12.

[bib1.bib36] Rustin M, Janssens S, Buys N, Gengler N (2009). Multi-trait animal model estimation of genetic parameters for linear type and gait traits in the Belgian warmblood horse. J Anim Breed Genet.

[bib1.bib37] Tsuruta S, Misztal I, Lawlor TJ (2005). Changing definition of productive life in US Holsteins: effect on genetic correlations. J Dairy Sci.

[bib1.bib38] Vanderick S, Troch T, Gillon A, Glorieux G, Gengler N (2014). Genetic parameters for direct and maternal calving ease in Walloon dairy cattle based on linear and threshold models. J Anim Breed Genet.

[bib1.bib39] Van Pelt ML, Meuwissen TH, Dejong G, Veerkamp RF (2015). Genetic analysis of longevity in Dutch dairy cattle using random regression. J Dairy Sci.

[bib1.bib40] Van Pelt ML, Dejong G, Veerkamp RF (2016). Changes in the genetic level and the effects of age at first calving and milk production on survival during the first lactation over the last 25 years. Animal.

[bib1.bib41] Varona L, Misztal I, Bertrand JK (1999). Threshold-linear versus linear-linear analysis of birth weight and calving ease using an animal model: II. Comparison of models. J Anim Sci.

[bib1.bib42] Weigel K (2006). Genetics of longevity and productive life. Adv Dairy Technol.

[bib1.bib43] Zavadilovà L, Zink V (2013). Genetic relationship of functional longevity with female fertility and milk production traits in Czech Holsteins. Czech J Anim Sci.

